# Distribution of Carcinogenic Human Papillomavirus Genotypes and Association to Cervical Lesions among Women in Fez (Morocco)

**DOI:** 10.1371/journal.pone.0146246

**Published:** 2016-01-05

**Authors:** Tiatou Souho, Hinde El Fatemi, Safae Karim, Karima El Rhazi, Chahrazed Bouchikhi, Abdelaziz Banani, Moulay Abdelilah Melhouf, Mohamed Benlemlih, Bahia Bennani

**Affiliations:** 1 Laboratoire de Microbiologie et de Biologie moléculaire, Faculté de Médecine et de Pharmacie de Fès (FMPF), Université Sidi Mohammed Ben Abdellah (USMBA), Fez, Morocco; 2 Laboratoire de Biotechnologies, Faculté des Sciences Dhar El Mahraz, USMBA, Fez, Morocco; 3 Laboratoire Central d'Analyse Médicale, CHU Hassan II, Fez, Morocco; 4 Laboratoire d'épidémiologie, Recherche Clinique et Santé Communautaire, FMPF, USMBA, Fez, Morocco; 5 Service de Gynecologie-Obstetrique I, CHU Hassan II, Fez, Morocco; 6 Service de Gynecologie-Obstetrique II, CHU Hassan II, Fez, Morocco; 7 Equipe micro-organismes, génomique et Facteurs Oncogènes, Laboratoire de Pathologie Humaine, Biomédecine et Environnement, FMPF, USMBA, Fez, Morocco; State University of Maringá/Universidade Estadual de Maringá, BRAZIL

## Abstract

**Objectives:**

To determine the distribution of cervical high-risk human papillomavirus genotypes and their association to cellular abnormalities in women from Fez and its neighborhood.

**Methods:**

Women attending the Hassan II University Hospital for cervical pap smears were recruited after an informed consent. Interviews and two cervical samples were performed for each woman. Cervical samples were used for cytological analysis and HPV DNA detection. HPV was typed using a method based on multiplex PCR with fluorescently labeled specific primers followed by capillary electrophoresis. The study was approved by the ethics committee of the Faculty of Medicine and Pharmacy of Fez.

**Results:**

The HPV prevalence in the studied population was 43.1% and the most prevalent types were HPV 53 (23 cases); HPV 16 (20 cases); HPV 35 (18 cases); HPV 51 (10 cases) and HPV 56 (7 cases). From the 619 confirmed pap smears, 20% were abnormal. The cytological abnormalities were significantly associated to HPV infection, women age, number of pregnancies and parity (p < 0.05).

**Conclusion:**

More attention should be given to HPV in Morocco because it represents an important public health concern. The distribution of carcinogenic HPV types in the studied population is different from the data in other regions but epidemiological studies in other Moroccan regions are required.

## Introduction

Human papillomavirus (HPV) is a group of double-stranded circular DNA virus that affects human epithelia including ano-genital mucosa. Most genital HPV infections remain asymptomatic and undergo spontaneous clearance without leaving any lesion. However, in some cases, persistent infection by at least one high-risk HPV genotype is responsible for precancerous lesions that can evolve to invasive cervical cancer [1, 2].

Cervical cancer prevention programs in western countries allowed a decrease in the incidence of cervical cancer cases and cervical cancer related deaths [3–7]. In contrast, developing countries are still showing relatively high prevalence rates of HPV infections and deaths related to this cancer. In addition, there is a lack of data regarding the distribution of high-risk HPV types especially in Africa. In many countries including Morocco, the lack of epidemiological data on high-risk HPV genotypes limits the implementation of cervical cancer prevention programs [8–10].

In the particular context of Morocco, little is known about the burden of HPV and the distribution of high-risk HPV types. However, some studies have been made and the prevalence of HPV infections in Moroccan women has been determined in some regions [11–13]. Two distinct studies have even evaluated the distribution of some HPV types (HPV 16, 18, 31, 33, 35, 45 and 59) in women attending the Mother and Children department of Ibn Sina Hospital or in cervical carcinoma biopsies collected in the Oncologic Center in Casablanca [12, 13]. A high prevalence of HPV in Fez was reported but the distribution of HPV types is unknown [11].

The aims of this study were i) to evaluate the prevalence of HPV infection in a population of women who consulted the University Hospital of Fez; ii) to determine the distribution of HPV genotypes using a newly developed HPV genotyping method that targets a total of sixteen HPV types including all the confirmed high-risk types; iii) to determine the risk factors associated to HPV infections or cellular abnormalities.

## Materials and Methods

### Ethic statement

All the recruited women were informed about the study objectives, methods and outcomes. Interviews and tests were performed only for consenting women. Due to the high rate of illiteracy, verbal consents were accepted and mentioned on the interview forms. All the participating women were assigned a code which is used to identify samples without individual information. All the procedures in this study were approved by the IRB of the Hassan II University Hospital, Fez.

### Study population

Women were recruited from November 2013 through December 2014 in the Laboratory of Anatomopathology of Hassan II University Hospital in Fez, Morocco. These women were referred by gynecologists (from different primary health care centers in the region) for cervical smears. A face-to-face interview was performed to collect socio-demographic and sexual behavior data from consenting women. The questions included age, educational level, profession, marital status, area of residence, number of sex partners, use of contraception, history of gynecological diseases, menopause, number of pregnancy and parity.

### Samples collection and DNA extraction

Two different cervical samples were performed successively. The first was performed with Cytoprep Brush and the cells were stored in CYTOfast preservative solution for liquid-based cytological smears. The smears were observed by three different pathologists and cellular lesions were classified according to the Bethesda 2001 system [14]. The second sample was stored in phosphate buffered saline (PBS) and used for DNA extraction. The DNA was extracted by cellular lysis by overnight incubation at 37°C with 50 μg/ml proteinase K. The lysis was stopped by enzyme deactivation with 10 minute incubation at 93°C. The DNA was then purified using a phenol/chloroform purification procedure. The DNA quality control was performed by a PCR test using specific primers for human beta-globin gene [15].

### HPV detection and typing

HPV DNA was detected using the broad spectrum primer set GP5+/GP6+ as described previously [16]. PCR products were revealed with electrophoresis on a 2% agarose gel using an ethydium bromide staining method. HPV positive samples were subjected to HPV typing using a recently developed method [17]. Briefly, HPV positive DNA samples were used in multiplex PCR with fluorescently labeled specific primers and multiplex PCR products were analyzed by capillary electrophoresis and fluorescence detection on ABI PRISM 3130 DNA analyzer. The method allows the specific identification of the twelve Group 1 carcinogenic HPV types (HPV 16, 18, 31, 33, 35, 39, 45, 51, 52, 56, 58 and 59) and four Group 2 carcinogenic types (HPV 53, 68, 73 and 82) [17, 18]. All the experiments for HPV detection or typing were performed in duplicate with negative and positive controls.

### Statistical analyses

Descriptive analyses were performed for all variables. Prevalence values of HPV infections and cytological abnormalities were calculated. The relationship between different variables and HPV infection or cellular abnormality was checked using Fisher exact test or Khi square test when applicable. After the univariate analysis, variables with p ≤ 0.20 were used in a stepwise selection procedure to determine the factors associated to HPV infection or cellular abnormalities in a logistic regression model. In all tests, the limit of significance was set to a P value of 0.05. All the statistical analyses were performed using SPSS statistics version 20.

## Results

A total of 633 participants coming from Fez and its neighborhood were included in this study. All the women were Moroccans, Muslims and aged 20 to 85 years old with a mean age of 46.55 (± 10.88) years. The women were referred from peripheral health care services where they first consulted for gynecological check-up (55.3%), control after the diagnosis of breast or ovary neoplasic lesions (17.2%), leucorrhoea (8.8%), metrorrhagia (7%), dysmenorrheal (4.1%), skin lesions (2.7%), sterility (2.4%), menstrual disorders (1,4%), prolapse (0,8%) and ectopic pregnancy (0,3%). All the women had never been tested for HPV infection and never received HPV vaccine. None of the women was an active smoker but 30.3% were passive smokers. Most of them (68%) were illiterate and 26.1% had already used oral contraception. Most of the women (93.3%) have only one lifetime sexual partner who is their husband and 70.9% had their first sexual intercourse under the age of 20 years old. Among these women, 49.5% bore more than three pregnancies and 25.7% experienced at least one miscarriage (Table 1).

**Table 1:** Description of the studied population.

Variable	N	%
**Age (years), n = 625**		
< 30	39	6.2
30–60	531	85
> 60	55	8.8
**Age at 1**^**st**^ **sexual intercourse (years), n = 611**		
≤ 20	433	70.9
> 20	178	29.1
**Education level, n = 618**		
Illiterate	420	67.9
Primary and secondary school	158	25.6
College	40	6.5
**Lifetime sexual partners number, n = 612**		
0	3	0.5
1	571	93.3
≥2	38	6.2
**Passive smoking n = 613)**		
Yes	186	30.3
No	427	69.7
**Oral contraception, n = 614**		
Yes	160	26.1
No	454	73.9
**Number of pregnancies, n = 618**		
≤ 3	312	50.5
> 3	306	49.5
**Parity, n = 619**		
≤ 3	362	58.5
> 3	259	41.5
**History of miscarriages, n = 615**		
Yes	158	25.7
No	457	74.3
**Menopause, n = 615**		
Yes	246	40
No	369	60

**Fig 1:**
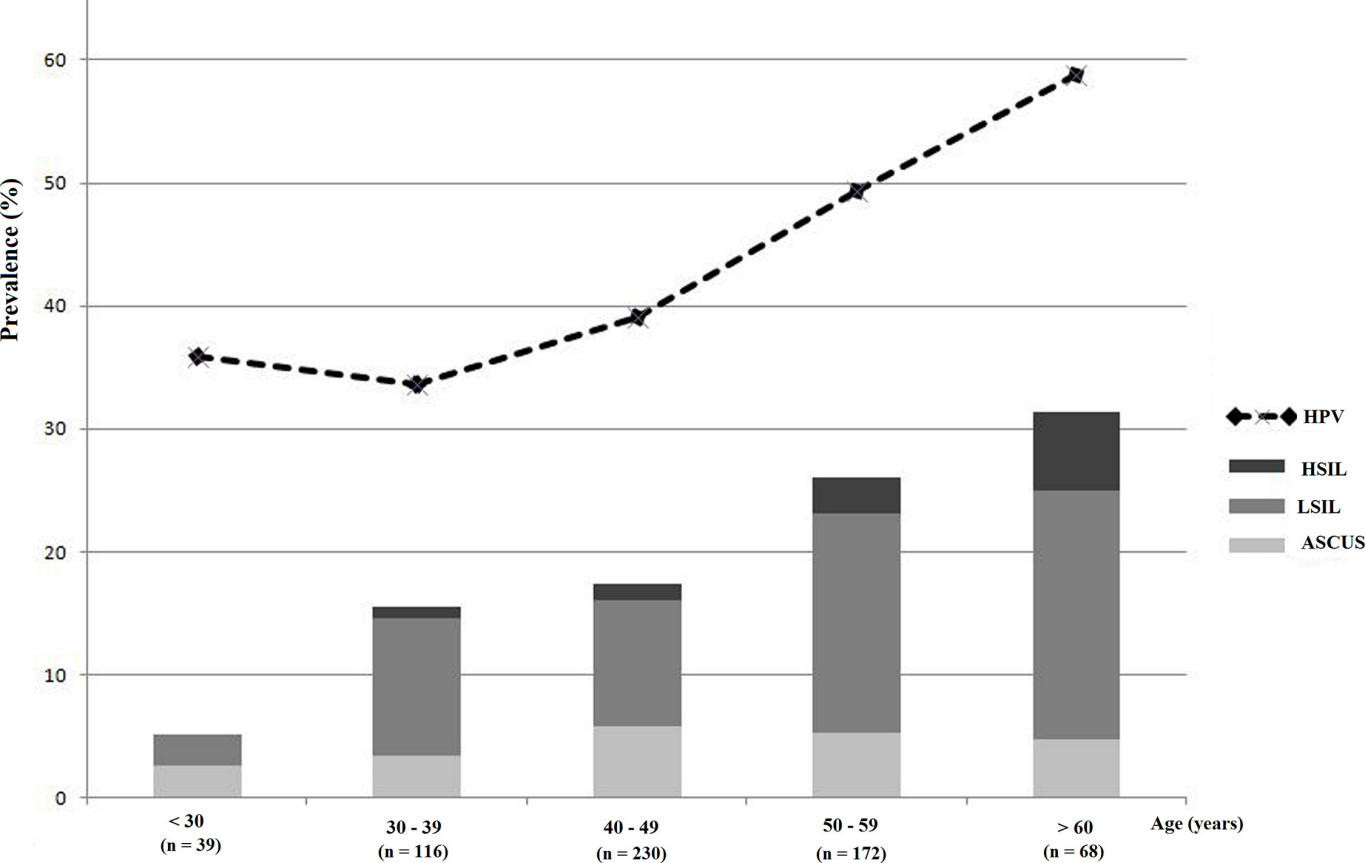
Prevalence of HPV and cytological lesions according to women age.

Among 633 samples, 14 did not contain enough cells for cytological interpretation. Cytological examination results were confirmed for 619 samples from which 495 (80%) showed a normal cytology and 122 abnormalities. The abnormal Pap smears include 30 (4.8%) atypical squamous cells of undetermined significance (ASCUS), 81 (13.1%) low-grade squamous intraepithelial lesions (LSIL) and 13 (2.1%) high-grade squamous intraepithelial lesions (HSIL). The prevalence of cellular abnormalities increases with women age: from 5.3% in women under 30 years old, it reaches a value of 31.3% in women above 59 years old (Fig 1). The highest age-specific prevalence values for LSIL (20.3%) and HSIL (6.3%) are recorded in this age group.

The quality of DNA extracted from all the 633 samples was good enough for HPV DNA detection and typing. The overall HPV prevalence in the studied population was 43.1%. HPV prevalence decreased from 35.9% in women under 30 years old to 33.6% in women between 40 and 49 years old and then increased progressively to reach the highest value (58.8%) in women of 60 years old and above (Fig 1). According to the cytological profile, HPV prevalence was 34.3%; 73.3%; 80.2% and 76.9% in women with normal cytology, ASCUS, LSIL and HSIL respectively.

Statistical analysis was made to determine factors associated with HPV infection. In the univariate analysis, HPV infection was found to be significantly associated with age, lifetime number of sexual partners and menopausal status (p < 0.05). The educational level, the age at first sexual intercourse, the use of oral contraceptives, passive smoking, history of miscarriage and the use of public bathrooms seemed not determinant in HPV acquisition (Table 2). The logistic regression analysis showed that HPV infection is significantly associated with menopause and lifetime number of sexual partners (Table 3).

**Table 2:** Correlations between HPV infection and other variables.

Variable*	N	HPV prevalence (%)
**Menopause,** p < 0.001		
No	369	36.31
Yes	246	52.44
**Age,** p < 0.001		
<30	39	35.9
30–60	531	41.6
>60	55	60
**Lifetime number of sexual partners,** p = 0.048		
1	571	41.5
≥ 2	38	57.9
**Oral contraception,** p = 0.085		
No	454	44.7
Yes	160	36.9
**Parity,** p = 0.106		
≤ 3	362	39.8
> 3	259	46.3
**History of miscarriage,** p = 0.151		
No	457	40.9
Yes	158	47.5
**Number of pregnancies,** p = 0.270		
≤ 3	312	40.4
> 3	306	44.8
**Use of public bathrooms,** p = 0.379		
No	224	41.52
Yes	387	43.15
**Passive smoking,** p = 0.408		
No	427	42.15
Yes	186	43.55
**Age at first sexual intercourse,** p = 0.503		
≤ 15	77	48.1
16–25	440	41.4
>25	94	44.7
**Education,** p = 0.783		
Illiterate	420	43.09
Literate	198	41.92

* P values were calculated using the Khi square test.

**Table 3:** Multivariate analysis of factors associated to HPV infections.

	P	Odds Ratio	95% CI
**Menopause**	**0.002**	**1.729**	**1.217–2.457**
More than one lifetime sexual partner	0.050	1.969	1.000–3.875
Oral contraception	0.330	0.823	0.556–1.218
Parity > 3	0.318	1.192	0.845–1.682
History of miscarriages	0.368	1.190	0.815–1.740

HPV positive samples underwent HPV typing using the method described above. Among HPV positive samples, 187 (68.5%) harbored low risk types and 86 (31.5%) harbored at least one carcinogenic HPV type. The five most frequent types are HPV 53, 16, 35, 51 and 56 which are identified respectively in 3.6%; 3.2%; 2.8%; 1.6% and 1.1% of cervical samples. Double infections were identified in 5 cases (0.8%). The distribution of HPV genotypes according to cytological results is presented in Table 4.

**Table 4:** HPV type-specific prevalence according to cytology.

Genotypes	Normal cytology (n = 495)	Abnormal cytology (n = 124)	All (n = 633)	P*
**Any HPV**	**170 (34.3)**	**97 (78.2)**	**273 (43.1)**	**< 0.001**
Single infections				
**HPV 16**	**10 (2)**	**9 (7.3)**	**20 (3.2)**	**0.002**
HPV 18	4 (0.8)	1 (0.8)	5 (0.8)	0.737
HPV 31	1 (0.2)	0	1 (0.2)	0.916
**HPV 35**	**8 (1.6)**	**10 (8.1)**	**18 (2.8)**	**< 0.001**
**HPV 45**	**0**	**3 (2.4)**	**3 (0.5)**	**0.008**
HPV 51	4 (0.8)	4 (3.2)	8 (1.3)	0.055
HPV 53	13 (2.6)	6 (4.8)	19 (3)	0.240
**HPV 56**	**1 (0.2)**	**4 (3.2)**	**5 (0.8)**	**0.007**
HPV 73	1 (0.2)	0	1 (0.2)	0.916
HPV 82	1 (0.2)	0	1 (0.2)	0.916
Double infections	4 (0.8)	1 (0.8)	5 (0.8)	0.737
**Low risk types**	**123 (24.8)**	**59 (47.6)**	**187 (29.5)**	**< 0.001**

* Comparison of type-specific prevalence between normal and abnormal cytology groups (p values were determined with Khi square or Fisher's exact test).

The relationship between cellular abnormality and other variables was also tested. In the univariate analysis, it was found that cytological abnormalities are significantly related to HPV positivity, women age, menopause, number of pregnancies, high parity and illiteracy (p < 0.05). The use of oral contraceptives, the age at first sexual intercourse, the lifetime number of sexual partners, the use of public bathrooms, passive smoking and history of miscarriage were not statistically related to cytological abnormalities (Table 5). After logistic regression analysis, cytological abnormalities were significantly associated to HPV infection and menopause. The odds-ratio and 95% confidence intervals are given in Table 6.

**Table 5:** Correlations between cytological abnormalities and other variables.

Variable*	N	Cytological Abnormalities (%)
**HPV,** p < 0.001		
Negative	352	7.7
Positive	267	36.3
**Menopause,** p < 0.001		
No	363	12.1
Yes	238	31.1
**Age,** p = 0.003		
< 30	38	5.3
30–60	522	19.9
> 60	51	33.3
**Education,** p = 0.039		
Illiterate	409	22.00
Literate	195	14.9
**Parity,** p = 0.048		
≤ 3	353	17.3
> 3	252	23.8
**Number of pregnancies,** p = 0.049		
≤ 3	303	16.8
> 3	301	23.3
**Oral contraception,** p = 0.053		
No	442	21.7
Yes	158	14.6
**Passive smoking,** p = 0.367		
No	418	18.9
Yes	181	22.1
**Use of public bath rooms,** p = 0.426		
No	219	20.55
Yes	378	19.58
**History of miscarriages,** p = 0.468		
No	445	19.1
Yes	156	21.8
**Lifetime number of sexual partners,** p = 0.534		
1	558	19.5
≥2	38	23.7
**Age at first sexual intercourse,** p = 0.958		
≤ 15	73	19.2
16–25	430	20.2
>25	94	19.1

* P values were calculated using the Khi square test.

**Table 6:** Multivariate analysis of factors associated to cytological abnormalities.

Factors	P	Odds Ratio	95% CI
**HPV positivity**	**< 0.001**	**6.251**	**3.852–10.142**
**Menopause**	**< 0.001**	**2.616**	**1.665–4.112**
Illiteracy	0.282	0.755	0.453–1.260
Number of pregnancies > 3	0.348	1.245	0.787–1.970

Type-specific prevalence in abnormal smears were compared to those in women with normal cytology. The prevalence of HPV 16, 35, 45 and 56 were significantly higher in women with abnormalities (Table 4). When the comparison is made in women under 40 years old, only HPV 35 showed a significantly high prevalence in abnormalities (p = 0.007). Low-risk HPV types taken together also show a significantly high prevalence in cellular abnormalities.

## Discussion

The studied population is not representative of the whole female population in Morocco. However, the results provide a first insight on the distribution of mucosal high-risk HPV types in women from Fez. A relatively high HPV prevalence (43.1%) was recorded confirming the value of 42.5% obtained in the previous study conducted on women with normal cytology in the period between February 2007 and December 2008 in the same region [11]. Most HPV infections are related to low risk types but the five most prevalent types are HPV 53, 16, 35, 51 and 56. HPV 18 has a low prevalence and comes only at the sixth place.

HPV prevalence and genotype distribution in the present study are different from the results obtained in women attending the department of children and mothers’ pathology of Ibn Sina hospital in Rabat where HPV prevalence was 15.7% with HPV 16 and 18 as the most prevalent types [12]. It is important to mention that the study performed in Rabat used a genotyping method that identifies only six types (HPV 16, 18, 31, 33, 35 and 45). Maybe the distribution of HPV types in this population could be readjusted if the typing method included all the high-risk types. It is also possible that the distribution of HPV types is not the same in these two cities. Two studies evaluated the distribution of HPV types in cervical carcinoma biopsies in Morocco. The first study explored the distribution of HPV 16, 18, 31, 33, 45, and 59 in 89 biopsies obtained from an oncologic center in Casablanca and the second one concerned 129 biopsies collected from Rabat and Casablanca [13, 19]. In both studies, HPV 16 and 18 were the most prevalent types and HPV 53 was not recorded.

The HPV infection profile in the studied population shows some similarities with HPV epidemiology in many other regions. Among these similarities are the high HPV prevalence and the bimodal shape of the HPV age-specific prevalence. In effect, high HPV prevalence rates were recorded in countries such as Australia (38.7); Cameroon (38.5%); Gabon (46%); Mexico (67.1%) and Saudi Arabia (31.6%) [20–24]. Moreover, like in this study, it was shown in many developing countries that the age specific HPV prevalence is bimodal with a first peak in young women and a second one in women above 50 years old [11, 25, 26].

The most prevalent genotype in this study is HPV 53. This genotype was also reported as the most prevalent type in Gabon [24]. The relatively low prevalence of HPV 16 and 18 is also reported in several studies. In a study performed in Saudi Arabia, the most prevalent types were HPV 68/73 followed by HPV 6 and 18 [27]. In several studies, HPV 16 is the leading type but HPV 18 does not come right in the second place. In China, HPV 16 is followed by HPV 52 and 58 [28, 29]. HPV 16 is followed by HPV 51 and 53 in Australia but it is followed by HPV 58 and 18 in Tunisia [23, 30]. All these data show the low prevalence of HPV 16 and 18 in women with normal cytology in comparison to cervical cancer cases. In effect, around 70% of cervical cancer cases are associated to these two genotypes [2, 13, 18].

The comparison between normal and abnormal pap smears regarding the distribution of HPV types shows clearly the difference between HPV 53 and the group 1 carcinogenic types. In effect, despite the high prevalence of HPV 53, it was not associated with cellular abnormalities whereas HPV 16, 35, 45 and 56 showed significant relationship with abnormalities. However, HPV 53 should be considered in future cervical cancer prevention programs in Morocco because molecular evidence of HPV 53 carcinogenicity has been provided [31]. Furthermore, the results in this study show that women infected with HPV 35 should be given particular attention because this genotype is associated to cellular abnormalities even in women under 40 years old.

The association of HPV infections with cytological abnormalities is highly significant. This shows that HPV infections in the studied region constitute a real public health concern. In effect, even if invasive cervical cancer cases are not recorded in this study, intra epithelial lesions of high significance were recorded. Among women with HSIL, the worst abnormality recorded in this study, HPV DNA was detected in 76.9% cases. This value is lower than HPV prevalence in HSIL cases in Africa (85%), Asia (78%), Europe (88%), Latin America (85%) and North America (86%) [32]. However, this value is higher than the 57% obtained in Bhutan [33].

The logistical regression models show clearly that HPV infections are responsible for cervical abnormalities. Increased number of pregnancies and menopause are both significantly related to HPV infections and cervical abnormalities. Most of women (93.3%) in the present study have only one lifetime sexual partner but women who had two or more lifetime sexual partners have an increased risk of HPV infection. However, the lifetime number of sexual partners was not associated to cellular abnormalities. This result shows that having two or more sexual partners increases the risk of HPV infection but it is not responsible for HPV persistence and cellular transformation. Cellular lesions result from HPV persistence after escaping from the immune system which is related to the hormonal balance. Pregnancy and menopause are responsible for many psychological, physiological and hormonal changes that can weaken the immune system [34]. The reduced immunity not only allows HPV infection but also constitutes a favorable medium for HPV persistence and cellular lesions appearance.

## Conclusion

Based on the results of this study, it is clear that HPV is a real public health concern in Morocco. A high rate of women harbor HPV infections and some of them even present precancerous lesions. The distribution of carcinogenic HPV genotypes in the studied region is very specific and shows that more epidemiological investigations are required. The next step could be the evaluation of HPV genotypes distribution in the general population of women from all the regions in Morocco and the distribution of HPV genotypes in cervical cancer cases collected from all the oncologic centers in Morocco.

Even if the studied population does not represent the whole Moroccan population, the results show that the future HPV prevention programs should be based on the epidemiological profile of HPV infections in the whole country. Nonetheless, women education, HPV screening in old women and appropriate monitoring of pregnant women could be helpful in preventing cellular lesions development. Finally, women in menopause should be given a particular attention.
